# Potential mechanisms of acupuncture in enhancing cerebral perfusion of ischemic stroke

**DOI:** 10.3389/fneur.2022.1030747

**Published:** 2022-10-28

**Authors:** Lu Wang, Xin-Tong Su, Yan Cao, Na-Na Yang, Xiao-Wan Hao, Hong-Ping Li, Qing-Yong Wang, Jing-Wen Yang

**Affiliations:** School of Acupuncture-Moxibustion and Tuina, International Acupuncture and Moxibustion Innovation Institute, Beijing University of Chinese Medicine, Beijing, China

**Keywords:** acupuncture, ischemic stroke, cerebral perfusion, hemodynamics, vasoactive substances, angiogenesis, microcirculation

## Abstract

Ischemic stroke is the predominant cause of long-term disability and death worldwide. It is attributable to the sudden interruption of regional cerebral blood flow, resulting in brain cell death and neurological impairment. Acupuncture is a widely used adjuvant treatment for ischemic stroke in China and shows promising efficacy in clinical practice. This review mainly focused on the evidence to illustrate several possible mechanisms of acupuncture therapy on cerebral perfusion in ischemic stroke. Studies have shown that acupuncture is probably effective in the enhancement of cerebral perfusion after ischemic stroke. It promotes the improvement of hemodynamics, the release of vasoactive substances, the formation of new blood vessels, as well as the restitution of microcirculation. Multiple factors may contribute to the variability in acupuncture's therapeutic effects, including the acupoint selection, stimulation frequency and intensity, and retaining needle time. Acupuncture has the potential to become a non-pharmacological adjuvant approach to enhance cerebral perfusion in ischemic stroke. Future studies are required to gain our insight into acupuncture as well as accelerate its clinical translation.

## Introduction

Ischemic stroke is a significant cause of morbidity and mortality worldwide and usually occurs when an artery supplying the brain becomes occluded (1). The substantial reduction of blood flow brings about the insufficient delivery of glucose and oxygen to the affected tissues, which ultimately results in the death of brain tissue and focal neurological deficits. Brain damage after ischemic stroke can be limited by rescuing the ischemic penumbra, which is severely hypoperfused and hypoxic, at-risk but not yet infarcted tissue (2). Evidence-based treatments to salvage the penumbra involve restoring blood flow as early as possible; otherwise, over time, the penumbra evolves into a core of irreversibly damaged tissue until it disappears completely. Therefore, timely revascularization therapies comprising intravenous thrombolysis and mechanical thrombectomy are the effective primary treatments for early ischemic stroke and are beneficial to improve the prognosis of neurological function, which are recommended by the current clinical guidelines (3, 4). However, their use is subject to a number of limitations, including the short time window, different approval restrictions, and many contraindications (2, 5).

Several non-pharmacological interventions, such as decompressive surgery (6), normobaric oxygen therapy (7), hypothermia (8), transcranial laser treatment (9), and sensory stimulation (10), showed great promise to slow down the demise of the penumbra as an adjunct to intravenous thrombolysis and endovascular thrombectomy and to improve functional outcomes. By constraining infarct growth, these interventions might also make patients generally excluded from intravenous alteplase and endovascular therapy eligible for this treatment, thereby increasing the number of treated patients, not only within but also beyond the currently approved time window (11). Acupuncture, as the primary method of treating diseases in Traditional Chinese Medicine for over 3,000 years, was a frequently applied non-pharmacological therapy that was claimed to be effective in treating strokes in many hospitals in China. Experimental studies showed its potential beneficial effects for ischemic stroke rehabilitation *via* benign regulation of oxidative stress (12), glutamate excitotoxicity (13), inflammation (14), apoptosis (15), and autophagy (16). Of note, increasing studies have indicated that acupuncture seems to be a helpful treatment for cerebral blood flow (CBF) enhancement following an ischemic stroke. However, the underlying mechanisms have not been well-understood to date. In this review, we focused on the evidence to illuminate how acupuncture had beneficial effects on CBF and its underlying mechanisms.

## Materials and methods

### Search strategy

Relevant studies were retrieved from PubMed database from 2000 to the present. Search terms consisted of three groups: interventions [“acupuncture” OR “electroacupuncture”], diseases [“ischemic stroke” OR “cerebral ischemia” OR “cerebral infarction”], and indicators [“cerebral perfusion” OR “cerebral blood flow” OR “collateral circulation” OR “brain circulation” OR “microcirculation” OR “hemodynamics” OR “angiogenesis” OR “blood viscosity” OR “blood flow velocity” OR “vasoactive substances” OR “vascular reactivity”].

### Study selection

**Figure 1:**
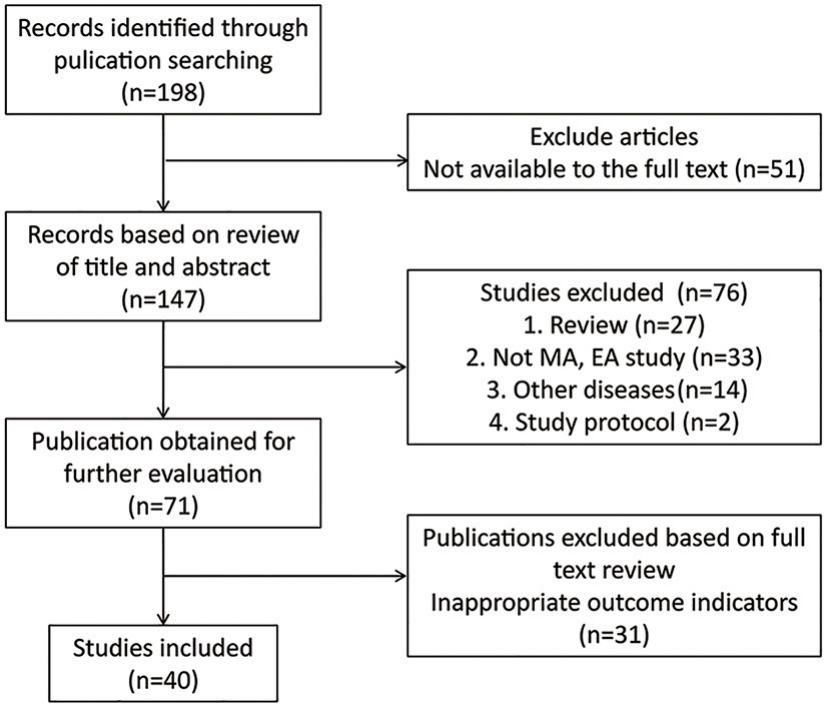
Flow chart of study selection. MA, manual acupuncture; EA, electroacupuncture.

We found 198 potentially relevant literatures *via* searching on an online database and of these articles, we excluded 158 articles for the following reasons: (1) full text was not available; (2) review; (3) studies that did not use manual acupuncture (MA) and electroacupuncture (EA); (4) other diseases; (5) study protocol; and (6) inappropriate outcome indicators. At last, 40 articles were included in this study. A flow chart of the search and filter process is shown in Figure 1.

## Acupuncture mechanisms on cerebral perfusion in ischemic stroke

### Acupuncture modulates cerebral hemodynamics

During ischemic stroke, the CBF decreases and the autoregulation of the cerebral vascular system is damaged, leading to cerebral ischemia and hypoxia, and this may lead to a poor prognosis. Accumulating evidence showed that acupuncture had a positive impact on cerebral hemodynamics. A randomized controlled trial showed that acupuncture significantly increased CBF velocity in patients with ischemic stroke, and the increase continued 5 min after needles were removed (17). Acupuncture elevated mean blood flow velocity and maximum peak flow speed, and lowered the vascular resistance index in patients with ischemic stroke (18). In addition, single-photon emission computed tomography was used for comparison brain perfusion images before and after acupuncture in patients with middle cerebral artery occlusion (MCAO). The results suggested that acupuncture could enhance regional CBF, peculiarly in the low perfusion area around the ischemic core, ipsilateral, or contralateral sensorimotor area (19). Another study reported that acupuncture was able to reduce higher shearing rate and lower shearing rate of whole blood viscosity, plasma viscosity, hematocrit, and profibrin in patients with ischemic stroke (20). Animal experiments showed similar results. After 15 min of MCAO in monkeys, EA for 1 h enhanced the local CBF in the striatum significantly (21). EA was administered for 15 min beginning 2 h after unilateral local ischemic infarction of the primary motor cortex was established in mice. The results revealed that EA rescued stroke-induced impairment of blood perfusion and neuronal activity in the contralateral primary motor cortex and primary sensory cortex (22). Moreover, EA for 20 min accelerated the CBF in the middle cerebral artery area of rats with bilateral common carotid artery occlusion (23). Likewise, acupuncture for 5 s recovered regional CBF in the ischemic cortex of MCAO rats (24) (Table 1). These findings suggest that acupuncture can be effective in enhancing cerebral hemodynamics by regulating blood flow velocity, vascular resistance index, blood viscosity during ischemic stroke.

**Table 1:** Acupuncture modulates cerebral hemodynamics.

**Subjects**	**Acupoint**	**Method**	**Frequency intensity**	**Duration**	**Treatment course**	**CBF detection technology**	**Effects of acupuncture**	**References**
Patients with ischemic stroke	LR3, LR4, SJ5, GB34	MA	-	20 min	Only once	TCD	Vm ↑	(17)
Patients with dysphagia in ischemic stroke	GV11/14/16/20/24/26	MA	Twist 30 s	30 min	5 days a week for 4 weeks	TCD	Vs, Vm ↑, RI ↓	(18)
Patients with MCAO	LI4/10/11/15/16, SJ5	MA	-	20 min	Only once	SPECT	CBF ↑	(19)
Patients with ischemic stroke	LI4/11/15, ST32/36/41, LR3, SJ5	EA	2 HZ, 2–6 mA	20, 40, or 60 min	10 days	rotary viscosity meter, capillary viscometers	whole blood low/high shear viscosity, plasma viscosity, hematocrit, fibrinogen ↓	(20)
MCAO monkeys	GV20/26	EA	−18/3.85 Hz, −7–8.8 mA, 6–8.8 mA	60 min	Only once	LDF	CBF ↑	(21)
Photochemical mice	GV14, GV20	EA	2/10 Hz, 1 mA	15 min	Only once	LSBFI	entire brain, contralateral, and S1 CBF↑	(22)
BCCAO rats	ST36, SP6	EA	2/15 Hz, 5 mA	30 min	Only once	LDF	CBF ↑	(23)
MCAO rats	GV26	MA	3 Hz	5 s	Two times a day for 3 consecutive days	LDF	CBF ↑	(24)
MCAO rats	PC6	EA	3 Hz	5, 60 or 180 s	Six times in 72 h	LDF	CBF ↑	(25)
MCAO rats	PC6	MA	twist 3 times/s	60 s	Five times in 60 h	LDF	CBF ↑	(26)
MCAO rats	GV11/16	EA	7 Hz, 6 mA	30 min	Pre-, intra- or post-ischemia	LDF	CBF ↑	(27)
MCAO rats	GV26, PC6, LU5, SP6, BL40	MA	Twist three times /min	5 s	Six times in 72 h	LDF	CBF ↑	(28)
MCAO rats	GV20/26, LI11, PC6, GB34, SP6	EA	5/20 Hz	5 min	Only once	LDF	CBF ↑	(29)
MCAO rats	GV20/26	EA	5/20 Hz, 1 mA	5, 15, 30, or 45 min	Only once	LDF	CBF ↑	(30)
MCAO rats	PC6	MA	1, 2, 3 Hz	5, 60, 180 s	Only once	LDF	CBF ↑	(31)

CBF, cerebral blood flow; MA, manual acupuncture; Vm, average blood flow velocity; TCD, transcranial Doppler ultrasound; Vs, maximum peak velocity; RI, Resistance index; MCAO, middle cerebral artery occlusion; SPECT, single-photon emission computed tomography; EA, electroacupuncture; LDF, laser Doppler flowmetry; LSBFI, laser speckle blood flow imaging; BCCAO, bilateral common carotid artery occlusion; LR3, Taichong; LR4, Zhongfeng; SJ5, Waiguan; GB34, Yanglingquan; GV11: Shendao; GV14, Dazhui; GV16, Fengfu; GV20, Baihui; GV24, Shenting; GV26, Shuigou; LI14, Binao; LI10, Shousanli; LI11, Quchi; LI15, Jianyu; LI16: Jugu; ST32, Futu; ST36, Zusanli; ST41, Jiexi; SP6, Sanyinjiao; PC6, Neiguan; LU5, Chize; BL40, Weizhong; ↑, upregulation; ↓, downregulation.

### Acupuncture modulates the release of vasoactive substances

Acetylcholine (ACh), considered a crucial mediator of cerebral vasodilation, can activate endothelial nitric oxide synthase (eNOS) by binding to M-type cholinergic receptors on the surface of the vascular endothelium and produce an appropriate amount of NO on smooth muscle cells to relax blood vessels. The central cholinergic system is susceptible to ischemia, and even mild hypoxia may impair the synthesis of ACh. 60 min post-MCAO, EA for 20 min prominently elevated the perfusion of the cerebral cortex. Cerebral perfusion began to increase at 10 s after EA stimulation, gradually enhanced during the stimulation process, and lasted until 20 min after the end of EA. Nevertheless, the increase of perfusion induced by EA was completely blocked by the M receptor blocker or eNOS gene knockout. It suggests that EA can alleviate the cerebrovascular damage in the acute phase of focal cerebral ischemia, at least in part due to the enhancement of cerebral perfusion in an ACh/eNOS-dependent manner (32). Consistent with this result, it was discovered that EA continuously and steadily built up CBF in the ischemic area, accompanied by an increase in the expression of choline acetyltransferase (ChAT) and five muscarinic receptor subtypes (33).

Angiotensin II (Ang II), as the main bioactive peptide of the brain renin-angiotensin system, plays an essential part in the pathophysiology of ischemic stroke. It has been declared that Ang II combined with Ang II type 1 receptor (AT1R), splits Phosphatidylinositol (4,5)-bisphosphate into diacylglycerol (DAG) and inositol triphosphate (IP3) that leads to the release of Ca^2+^ and mediates vasoconstriction. EA was found to reverse the upregulation of the IP3 signal transduction pathway *via* inhibiting Ang II binding to AT1R, which improved the blood supply of ischemic areas and had a beneficial effect on cerebral ischemia (34). Likewise, the other study found that EA effectively decreased the over-expression of DAG, IP3, and calmodulin in rats with acute cerebral infarction, improved cerebral autonomy movement, and alleviated cerebral vascular spasm (35).

**Figure 2:**
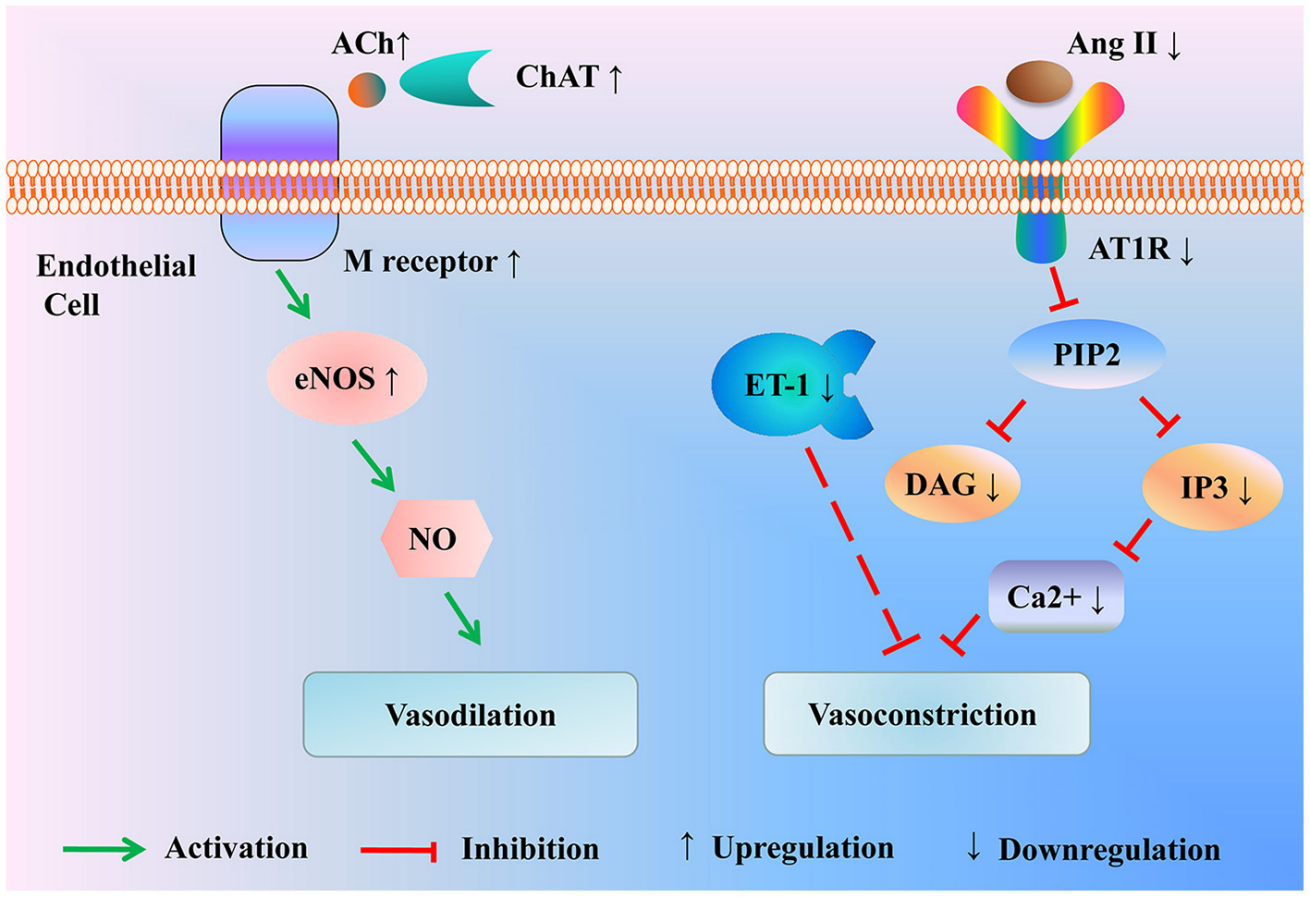
Modulation of the vasoactive substance by acupuncture for treating ischemic stroke. Acupuncture enhances the expression of ChAT to promote the activation of the ACh/eNOS signal pathway, resulting in vasodilation. Besides, acupuncture drops the level of Ang II and AT1R to inhibit the activation of the IP3 signal pathway, thus mitigating vasoconstriction. In the meantime, acupuncture alleviates the production of vasoconstrictor factor ET-1. ACh, Acetylcholine; ChAT, choline acetyltransferase; eNOS, endothelial nitric oxide synthase; Ang II, Angiotensin II; AT1R, Ang II type 1 receptor; PIP2, phosphatidylinositol (4,5)-bisphosphate; DAG, diacylglycerol; IP3, inositol triphosphate; ET-1, endothelin-1.

Moreover, endothelin-1 (ET-1) is by far the most powerful vasoconstriction peptide in both arteries and veins, which has a robust contractile effect on vascular smooth muscle. Scalp acupuncture inhibited the increase of peripheral plasma ET-1 in patients with cerebral infarction, contributing to the improvement of vascular elasticity and cerebral blood circulation as well as the reduction of the disability rate (36) (Figure 2 and Table 2).

**Table 2:** Acupuncture modulates the release of vasoactive substance.

**Subjects**	**Acupoint**	**Method**	**Frequency intensity**	**Duration**	**Treatment course**	**CBF detection technology**	**Effects of acupuncture**	**References**
MCAO mice	GV14/20	EA	2 Hz, 1 mA	20 min	Only once	LDF	ACh/eNOS↑, CBF↑	(32)
MCAO rats	GV14/20	EA	2/15 Hz, 1 mA	30 min	Only once	LDF	ChAT, five muscarinic receptor subtypes ↑	(33)
MCAO rats	GV26	EA	15 Hz, 1 mA	20 min	Only once	LDF	Ang II, AT1R, DAG, IP3, CaM ↓, CBF ↑	(34)
MCAO rats	GV26	EA	15 Hz, 1 mA	20 min	Only once	LSCM	Gq, DAG, IP3, CaM↓, CBF↑	(35)
Patients with cerebral infarction	LI4/11, ST34/36/41, SJ5, GB20, LR3	MA	twist once every 10 min	30 min	10 days	-	ET-1↓	(36)

CBF, cerebral blood flow; MCAO, middle cerebral artery occlusion; EA, electroacupuncture; LDF, laser Doppler flowmetry; ACh, Acetylcholine; eNOS, endothelial nitric oxide synthase; ChAT, choline acetyltransferase; Ang II, Angiotensin II; AT1R, AngII type 1 receptor; DAG, diacyl glycerol; IP3, inositol triphosphate; CaM, calcium/calmodulin-dependent protein; LSCM, laser scanning confocal microscope; MA, manual acupuncture; ET-1, endothelin-1; GV14, Dazhui; GV20, Baihui; GV26, Shuigou; LI4, Hegu; LI11, Quchi; ST34, Liangqiu; ST36, Zusanli; ST41, Jiexi; SJ5, Waiguan; GB20, Fengchi; LR3, Taichong; ↑, upregulation; ↓, downregulation.

Therefore, the potential efficacy of acupuncture in ischemic stroke may be its influence on the endothelial nitric oxide system and brain renin-angiotensin system, which can enhance cerebral perfusion by affecting cerebrovascular reactivity.

### Acupuncture modulates angiogenesis

The collateral circulation in the initial phase of ischemic stroke relies on the opening of a preexisting vascular network, while in the later period, it mainly depends on the formation of new blood vessels. Angiogenesis takes place within 12 to 24 h following ischemic insults and persists for at least 3 weeks. The central link of angiogenesis is the proliferation, migration, differentiation, and lumen formation of vascular endothelial cells (ECs). Endothelial progenitor cells (EPCs) primarily exist in the bone marrow (BM) and can enter the brain to differentiate into mature ECs, leading to neovascularization (37). EA promoted the increase of the number of EPCs in peripheral blood (PB) and BM at each phase of cerebral ischemia, modulating the mobilization, migration and homing of EPCs (38). It was observed that the ECs of MCAO rats began to proliferate within 24 h, reached the peak on the third day, and decreased on the 7th day. After EA, ECs proliferation accelerated to 12 h, and the number of ECs on the first, second, third, and 7th day was remarkably higher than that in MCAO rats (39). Angiogenesis is a dynamic and complex process engaged in a variety of cytokines and cellular components. In the microenvironment of vascular ECs, angiogenic factors and antiangiogenic factors are always keeping a dynamic balance and jointly regulate the formation of neovascularization.

#### Vascular endothelial growth factor (VEGF) pathway

VEGF is deemed to be the most critical factor in modulating angiogenesis. Recent studies have proved that acupuncture elevated the level of VEGF in plasma and cerebral ischemic tissue, which increased EPCs number and improved endothelial function in MCAO rats or patients with ischemic stroke (40–42). Moreover, the cerebral cortical miRNA profile in MCAO rats identified that the VEGF signaling pathway was most prominently affected by EA (43). The angiopoietin/ tyrosine kinase receptor 2 (Ang/Tie-2) system was considered a well-suited complement for VEGF: VEGF induced vascular budding while Ang/Tie-2 system promoted maturation of vessels (44). Endostatin is a vascular inhibitor, antagonizing the effect of VEGF (45). It was reported that EA up-regulated the expression of VEGF, Ang-1, Ang-2, and Tie-2 in the cortex round the ischemic necrotic region. Meanwhile, EA decreased the level of endostatin protein in the ischemic penumbra of focal cerebral ischemic rats (46, 47). Moreover, VEGF binds specifically to VEGF receptor-2 (VEGFR-2) on the surface of vascular ECs, activating intracellular signal transduction pathways and promoting neovascularization (48). Basic fibroblast growth factor (bFGF) encourages angiogenesis synergistically with VEGF. It was reported that EA enhanced the levels of VEGF, VEGFR2, and bFGF, thus inducing the growth of microvessels and the increase of regional CBF in the ischemic brain tissue (49). Another studies indicated that the classical Wnt/β-catenin signal pathway played crucial roles in maintaining BBB homeostasis and promoting vascularization by synergistic effects with angiogenesis-related factors. EA could activate the Wnt/β-catenin signaling pathway, thereby elevating the expressions of angiogenic factors including VEGF, bFGF, and Ang-2 and restoring blood perfusion in the ischemic zone (50). Besides, compared with the rats that received only thrombolysis 6 h after the embolic stroke model was established, the rats that received acupuncture for 30 min 2 h after stroke and before thrombolysis showed lower neurologic scores, less infarct volumes, and more expressions of VEGF and bFGF in ischemic areas of the cerebral cortex, indicating that acupuncture was able to prolong the time window of thrombolysis in cerebral infarction rats, which may be connected with the up-regulation of VEGF and bFGF expressions in ischemic cerebral cortex (51).

#### EphB4/EphrinB2 pathway

EphB4/EphrinB2 was reported to regulate the migration of ECs and angiogenesis by activation of Src and phosphoinositide 3 kinase (PI3K) signaling pathways. EA could accelerate capillary formation in the brain infarction area of MCAO rats through up-regulating EphB4 and EphrinB2 mRNA and increasing the level of Src and PI3K, manifesting that the formation of new vessels after EA was partly to regulated by EphB4/EphrinB2 mediated Src/PI3K signal pathway (52).

#### Stromal cell-derived factor-1 α (SDF-1α)/ CXC chemokine receptor 4 (CXCR4) pathway

SDF-1α is also considered an essential factor in angiogenesis by inducing the migration of EPCs. Following cerebral ischemia, the level of SDF-1α was reduced in BM and increased in PB. EA accelerated the formation of SDF-1α concentration gradient and increased the number of new blood vessels (38). Further study demonstrated that SDF-1α was bound to CXCR4 on the surface of EPCs (53). EA promoted cerebral angiogenesis *via* regulating SDF-1α/CXCR4 axis (54).

#### Apelin/APJ pathway

**Figure 3:**
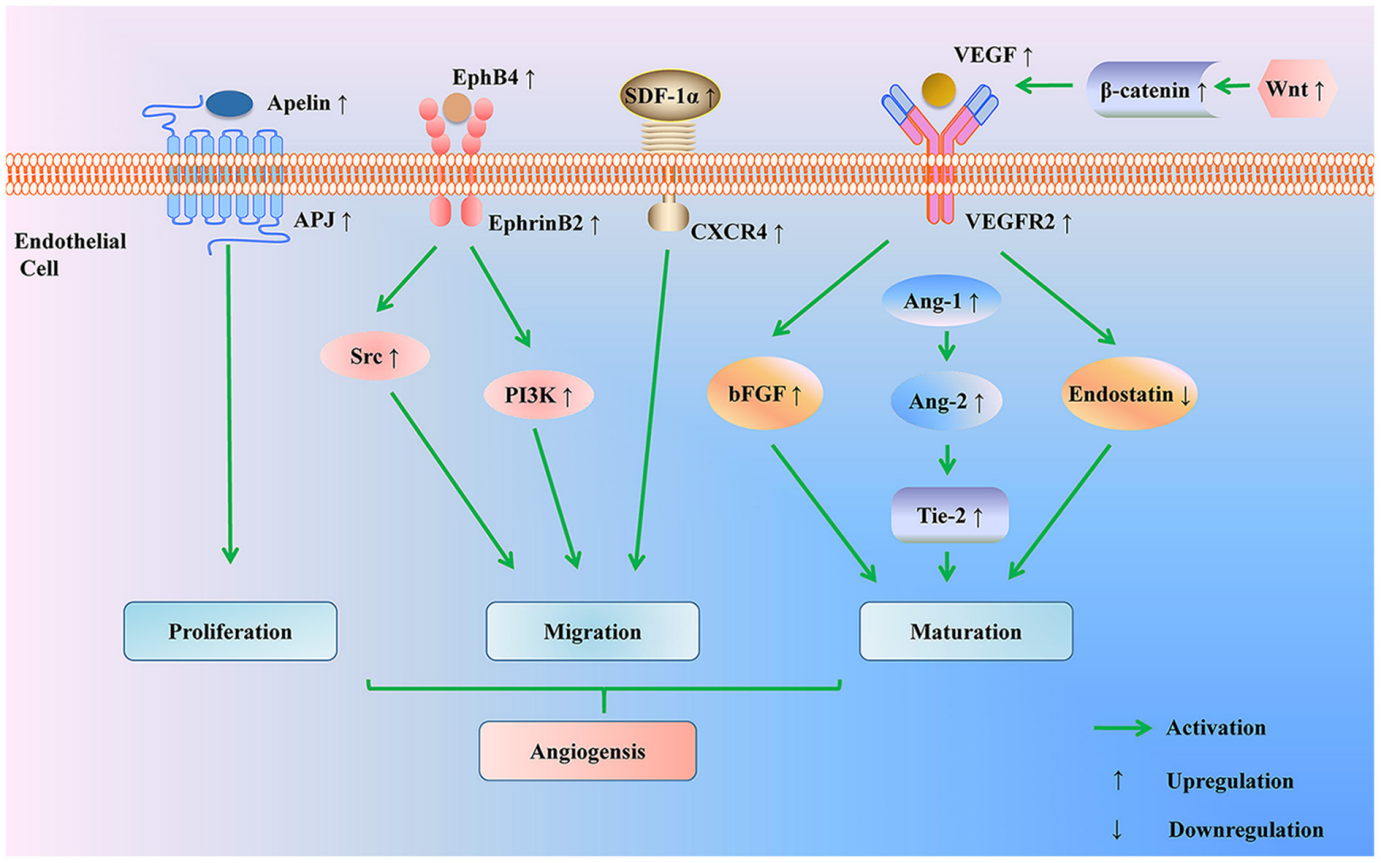
Regulation of angiogenesis pathway by acupuncture for treating ischemic stroke. Acupuncture promotes angiogenesis through multiple pathways: acupuncture up-regulates Apelin-APJ system to induce endothelial cells proliferation; acupuncture promotes endothelial cells migration by modulating the EphB4/EphrinB2 signaling pathway and SDF-1/CXCD4 axis; acupuncture activates the Wnt/β-catenin signaling pathway to elevate the expressions of angiogenic factors (VEGF, bFGF, Ang-1, Ang-2, and Tie-2) and to reduce the level of anti-angiogenic factor (endostatin). PI3K, phosphoinositide 3 kinase; SDF-1α, stromal cell-derived factor-1 α; CXCR4, CXC chemokine receptor 4; VEGF, vascular endothelial growth factor; VEGFR2, VEGF receptor-2; bFGF, basic fibroblast growth factor; Ang-1, Angiopoietin-1; Ang-2, Angiopoietin-2; tyrosine kinase receptor 2 (Tie-2).

Apelin-APJ system is involved in the proliferation of vascular smooth muscle cells and inhibits the high vascular permeability caused by VEGF (55). EA increased the expression of Apelin, APJ mRNA, and protein in the cerebral vascular endothelium of MCAO rats in 1, 9, 12, 24 h and 3, 7, 12 days, facilitating angiogenesis after cerebral ischemia (56) (Figure 3 and Table 3).

**Table 3:** Acupuncture modulates angiogenesis.

**Subjects**	**Acupoint**	**Method**	**Frequency intensity**	**Duration**	**Treatment course**	**CBF detection technology**	**Effects of acupuncture**	**References**
Patients with cerebral infarction	ST36/37, PC5, and PC6 on the left side	EA	2 Hz	20 min	Only once	-	ECs number ↑, VEGF ↑	(40)
MCAO rats	LI4, PC3, PC6, LI11	EA	20 Hz, 2–4 V	30 min	6, 24, 48, and 72 h after MCAO	-	VEGF ↑, microvascular number ↑	(41)
MCAO rats	GV20, GV26, ST36	EA	2/15 Hz, 1 mA	30 min	Once daily for 1, 2, 4, or 8 days	-	VEGF mRNA↑	(42)
MCAO rats	LI4	EA	40/60 Hz, 1.5 V	15 min	7 days	-	VEGF, Ang-1 ↑, Endostatin ↓	(46)
MCAO rats	GV26	EA	15 Hz, 0.1 mA	20 min	3, 6, 12, 24 h 3, 7, 12 d	-	Ang-1, Ang-2, Tie-2 ↑	(47)
MCAO rats	GV20, GV26, PC6	EA	2/20 Hz, 3–5 V	20 min	14 days	LDF	VEGF, VEGFR2, bFGF ↑	(49)
MCAO rats	GV20, GV26, PC6	EA	2/20 Hz, 3–5 V	20 min	14 days	-	VEGF, bFGF ↑	(57)
MCAO rats	GV26	EA	15 Hz, 0.1 mA	20 min	one time per day in 2, 3, 7, and 12 day	LDF	ECs number ↑, CBF in the ischemic boundary region ↑	(39)
MCAO rats	GV26, PC6	EA	2 Hz, 3 mA	1 min	1, 2, or 3 weeks	LDF	alter miRNA, CBF ↑	(43)
MCAO rats	GV26	EA	15 Hz, 1 mA	5 min	Only once	LDF	CBF on the infarct and non-infarct sides ↑, number of blood vessels ↑	(58)
MCAO rats	GV20	EA	3–15 Hz 2–4 mA	30 min	21 days	LDF	VEGF, FLK1, bFGF, Ang2↑, Wnt3a, β-catenin, cyclin D1↑	(50)
Embolic stroke rats	GV26, PC6	MA	Twist 1 min	30 min	Only once	-	VEGF, bFGF↑ endostatin↓	(51)
MCAO rats	GV4, GV9, GV14, GV20, GV26	EA	15 Hz	30 min	7 days	-	EphB4, EphrinB, Src, PI3K↑	(52)
MCAO rats	GV20, LI4, LR3	EA	2/20 Hz, 1 mA	30 min	7 days	-	SDF-1α ↑	(38)
MCAO rats	GV20, ST36	EA	40 Hz, 1–2 mA	20 min	14 days	**-**	SDF-1α ↑	(54)
MCAO rats	GV26	EA	15 Hz, 2 mA	20 min	1, 3, 6, 9, 12, or 24 h after MCAO; Once daily for 3, 7, or 12 days after MCAO	-	Apelin-APJ ↑	(56)

CBF, cerebral blood flow; EA, electroacupuncture; ECs, endothelial cells; VEGF, vascular endothelial growth factor; MCAO, middle cerebral artery occlusion; Ang-1, Angiopoietin-1; LDF, laser Doppler flowmetry; VEGFR-2, VEGF receptor-2; bFGF, basic fibroblast growth factor; PI3K, Phosphoinositide 3 kinase; SDF-1α, stromal cell-derived factor-1 α; ST36, Zusanli; ST37, Shangjuxu; PC5, Jianshi; PC6, Neiguan; LI4, Hegu; PC3, Quze; LI11, Quchi; GV20, Baihui; GV26, Shuigou; GV4, Mingmen; GV9, Zhiyang; GV14, Dazhui; LR3, Taichong; ↑, upregulation; ↓, downregulation.

The evidences above show that acupuncture can trigger angiogenesis through a variety of signaling pathways, thus having a positive impact on cerebral perfusion.

### Acupuncture modulates microcirculation

The primary structural and functional basis of the cerebral microcirculation is the blood-brain barrier, composed of microvasculature ECs sealed together by tight junctions, basement membranes, and astrocytes. After cerebral ischemia, the microvascular are usually narrowed or blocked by compression ascribed to the swelling of the foot process of astrocytes. Moreover, under the action of plasminogen activator inhibitor-1 secreted by vascular ECs, red blood cells, white blood cells, platelets, and fibrin are deposited in the narrow blood vessels, thus forming a microthrombus and blocking the microvessels. The microcirculation disturbance is a dominating cause of low perfusion and no reflow. As a consequence, the improvement of microcirculation is of great significance in the metabolism of tissue cells and the recovery of neurological function after ischemia.

When the brain is hypoxic-ischemic, glutamate produced by neurons combines with metabotropic glutamate receptors in astrocytes, inducing the expression of arachidonic acid cytochrome P450 epicytoxygenase (CYP2C11) through signal transduction. CYP2C11 catalyzes arachidonic acid to generate epicosane trienoic acids (EETs), that act on vascular smooth muscle, contributing to dilating microcirculatory vessels and increasing blood perfusion (59). CYP2C11 is a crucial enzyme in amino acid metabolism that indirectly reflects the ability of astrocytes to release EETs. EA raised blood flow in the pial meningeal microcirculation of MCAO rats by up-regulating the expression of CYP2C11 mRNA (60). Furthermore, acupuncture effectively dilated the diameter of microvessels and augmented the blood flow of the leptomeningeal microcirculation (61, 62) (Table 4). Therefore, it is reasonable to infer that acupuncture may enhance microcirculation through the regulation of key enzymes in cell metabolism, thereby improving neurological function prognosis in ischemic stroke.

**Table 4:** Acupuncture modulates microcirculation.

**Subjects**	**Acupoint**	**Method**	**Frequency intensity**	**Duration**	**Treatment course**	**CBF detection technology**	**Effects of acupuncture**	**References**
MCAO rats	PC6, LI11	EA	2/15 Hz, 1 mA	20 min	7 days	LDF	CYP2C 11 mRNA ↑ CBF ↑	(60)
MCAO rats	GV15/16/17, GB20	MA	Twist 1 min	15 min	14 days	LDF	Microcirculation ↑, Blood viscosity ↓	(62)
MCAO rats	PC6	MA	Twist 60, 120, 180 times /min	5, 60, or 180's	Only once	LDF, Microcirculation detector	CBF↑	(61)

CBF, cerebral blood flow; MCAO, middle cerebral artery occlusion; EA, electroacupuncture; CYP2C11, arachidonic acid cytochrome P450 epicytoxygenase; MA, manual acupuncture; LDF, laser Doppler flowmetry; PC6, Neiguan; LI11, Quchi; GV15, Yamen; GV16, Fengfu; GV17, Naohu; GB20, Fengchi; ↑, upregulation; ↓, downregulation.

## Factors associated with the effect of acupuncture on cerebral perfusion

### Acupoint selection

**Figure 4:**
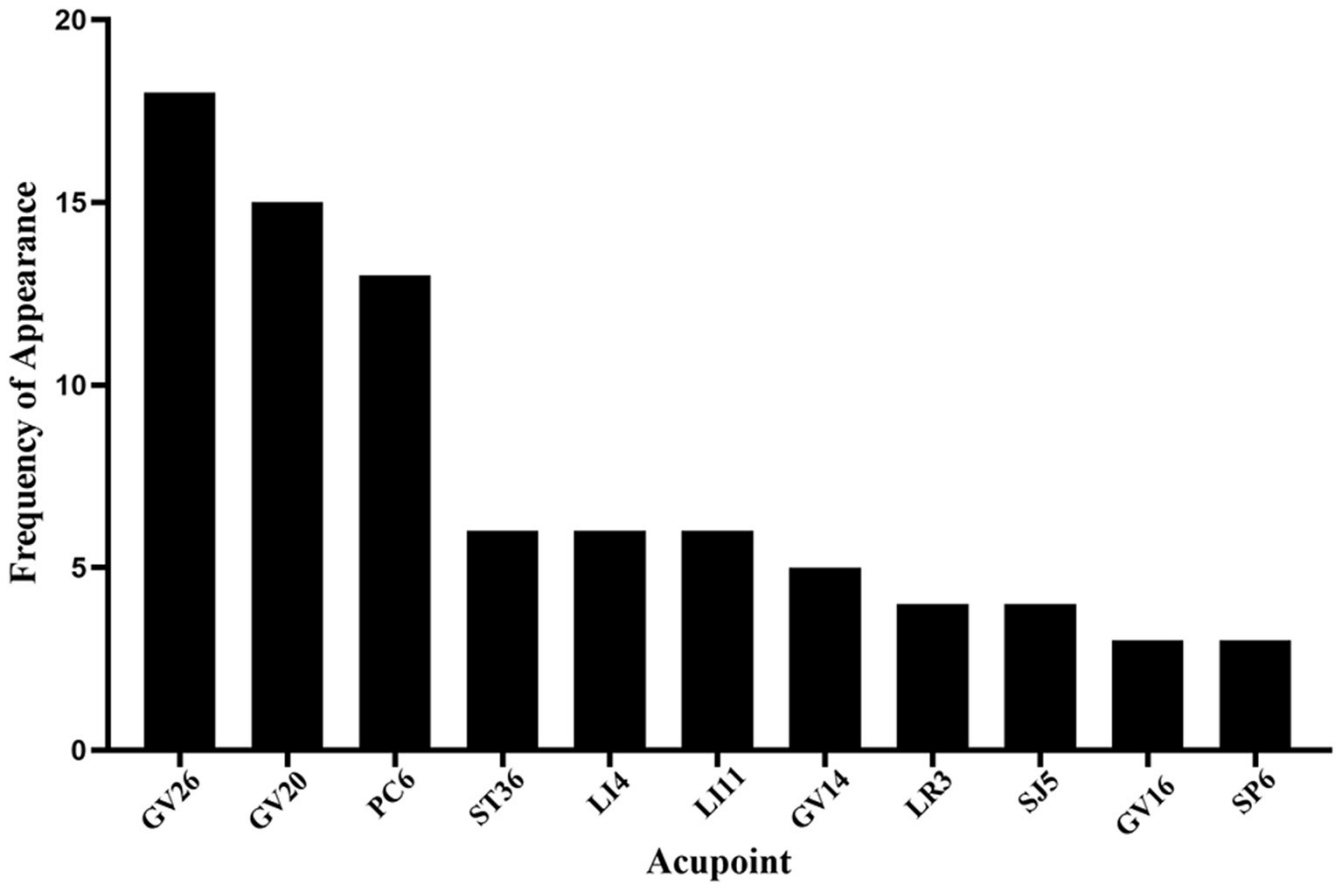
Frequency summary of individual acupoint. Only the acupoint that appears more than twice is shown. GV26: Shuigou; GV20: Baihui; PC6: Neiguan; ST36, Zusanli; LI4: Hegu; LI11: Quchi; GV14: Dazhui; LR3: Taichong; SJ5: Waiguan; GV16: Fengfu; SP6: Sanyinjiao.

**Figure 5:**
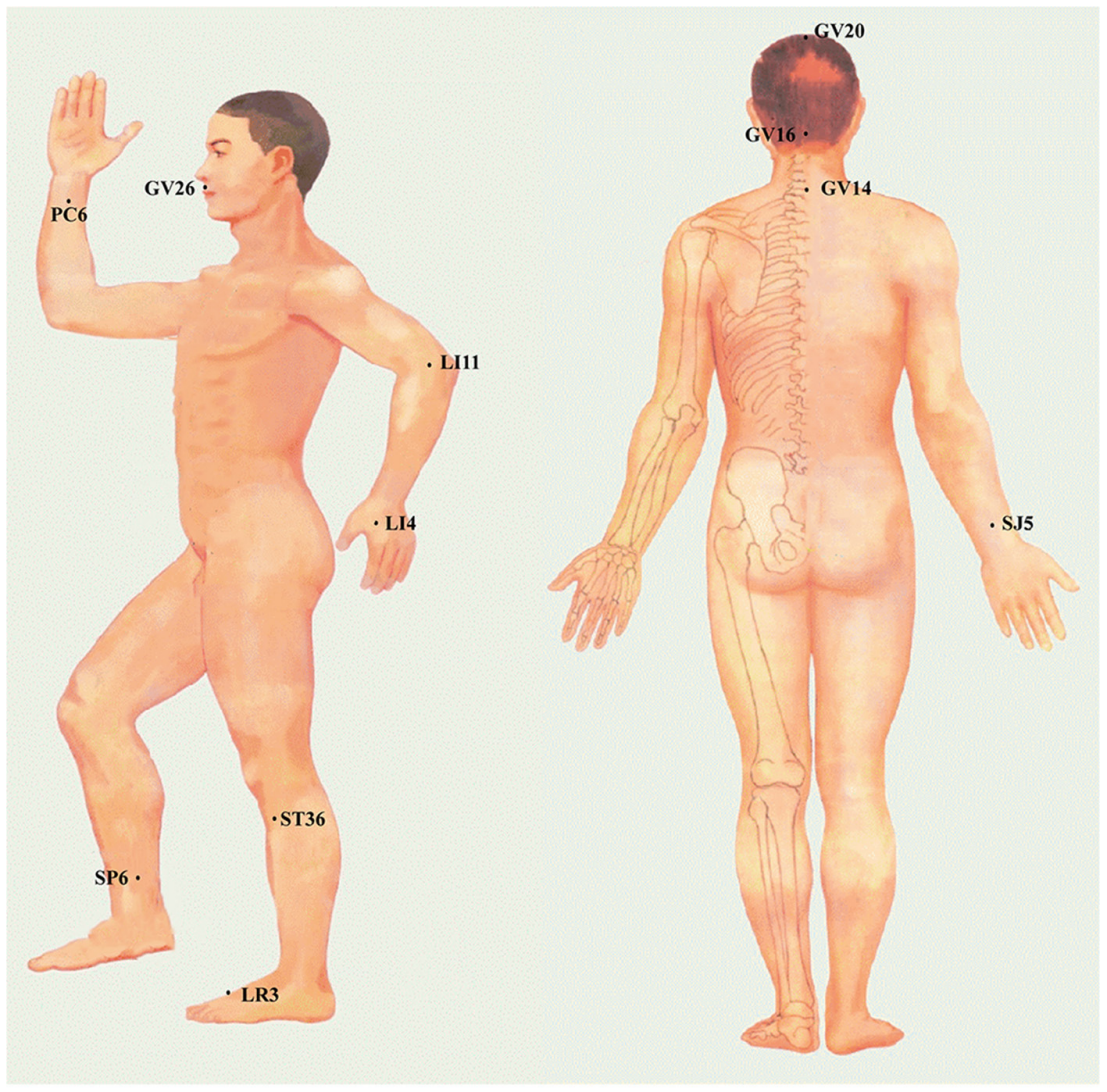
The specific location of acupoints is frequently applied to regulate CBF in the ischemic stroke.

Among the 40 articles included in this review, the three most frequently selected acupoints to include: (1) Shuigou (GV26), (2) Baihui (GV20), and (3) Neiguan (PC6) (Figures 4, 5). A study compared the difference of CBF regulation effect of Shuigou (GV26), Baihui (GV20), Neiguan (PC6), Quchi (LI11), Yanglingquan (GB34), and Sanyinjiao (SP6) acupoints on MCAO rats. The result showed that EA at Shuigou (GV26) and Baihui (GV20) exerted the best effects, which caused a striking increase in CBF, and the enhancement of blood perfusion was synchronized with EA (29). In another study, acupuncture was applied to Shuigou (GV26), Neiguan (PC6), Sanyinjiao (SP6), Chize (LU5), and Weizhong (BL40) acupoints in MCAO rats; the result identified that only Shuigou (GV26) and Neiguan (PC6) had a significant effect on restoring CBF (28). Besides, it was discovered that the pericardial meridian acupoint group [Quze (PC3) and Neiguan (PC6)] was notably superior to that of the large intestinal meridian acupoint group (Hegu (LI4) and Quchi (LI11)) in facilitating microangiogenesis (41).

### Stimulation frequency and intensity

Different stimulation frequency and intensity produced different effects on cerebral perfusion after ischemic stroke. EA at Shuigou (GV26) and Baihui (GV20) was given in diverse intensities (0–1.2 mA) and frequencies (1–100 Hz). The results were that the frequency of 5-20 Hz produced the best effect, which drastically conduced to the increase of blood perfusion and blood cell concentration (63). When rats were treated with EA at Shuigou (GV26) and Baihui (GV20) with the fixed frequency of 5/20 Hz, 0.6 mA stimulation did not spark off CBF alter, but the blood flow was considerably upregulated from 0.6 mA to 0.8 mA. When it changed to 1.0 mA, the CBF further increased twice as much as before EA, whereas the stimulation intensity of 1.2 mA maintained the same level. Consequently, the optimal intensity of EA stimulation at Shuigou (GV26) and Baihui (GV20) may range from 1.0 and 1.2 mA (63). The combination of varying acupuncture intensity and frequency may produce unlike therapeutic effects. The effects of diverse parameter combinations (0.4 mA, 5/20 Hz), (1.0 mA, 5/20 Hz), (1.0 mA, 70 Hz), on CBF were compared in MCAO rats receiving EA at Shuigou (GV26) and Baihui (GV20). The outcomes showed that the therapeutic effect of 1.0 mA, 5/20 Hz was strikingly better than that of the other two groups, resulting in a manifest increase in CBF (63).

Twisting technique was used in needling at Neiguan (PC6) with different frequencies (60, 120, and 180 times/minute). The neurological deficit score, leptomeningeal blood flow, microcirculation, and cerebral infarction rate were taken as effective indexes. The consequences revealed that the acupuncture parameter of the best acupuncture effect was 180 times/minute, in other words, fast frequency, which had apparent advantages in enhancing CBF and alleviating the rate of cerebral infarction (61).

### Retaining needle time

The optimal duration of acupuncture stimulation was of great importance to cerebral perfusion, infarction volume, neurological deficits degree, and mortality. In one study, EA was delivered to Shuigou (GV26) and Baihui (GV20) with sparse-dense wave (5/20 Hz) at 1.0 mA for 5, 15, 30, and 45 min, respectively. The consequences showed that 30 min of EA noticeably enhanced CBF, reduced the volume of cerebral infarction, and ameliorated the defect of neurological function. Although EA 45 min also elevated the CBF during MCAO, it brought about a worsening of mortality (30). Similar to this result, in the other study, acupuncture was applied to Neiguan (PC6) at a fixed frequency of 3 Hz (180 times/minute) with different durations, i.e., 5, 60 and 180 s. The results demonstrated that the therapeutic effect of acupuncture for 60 s was significantly better than that for 5 s and 180 s, with faster CBF, better recovery of neurological function, and smaller cerebral infarction volume (25).

So far, Shuigou (GV26), Baihui (GV20), and Neiguan (PC6) were the commonly selected acupoints and exerted the better effects on enhancing cerebral perfusion in ischemic stroke. EA at Shuigou (GV26) and Baihui (GV20) with appropriate intensity (1.0 mA), frequency (5/20 Hz), and retaining needle time (30 min) effectively increases the blood flow to the ischemic brain region. Neiguan (PC6) with appropriate twisting-rotating frequency (180 times/minute) and retaining needle time (60 s) showed the highest increase in CBF and the best protective effect on neurological function.

## Discussion

**Figure 6:**
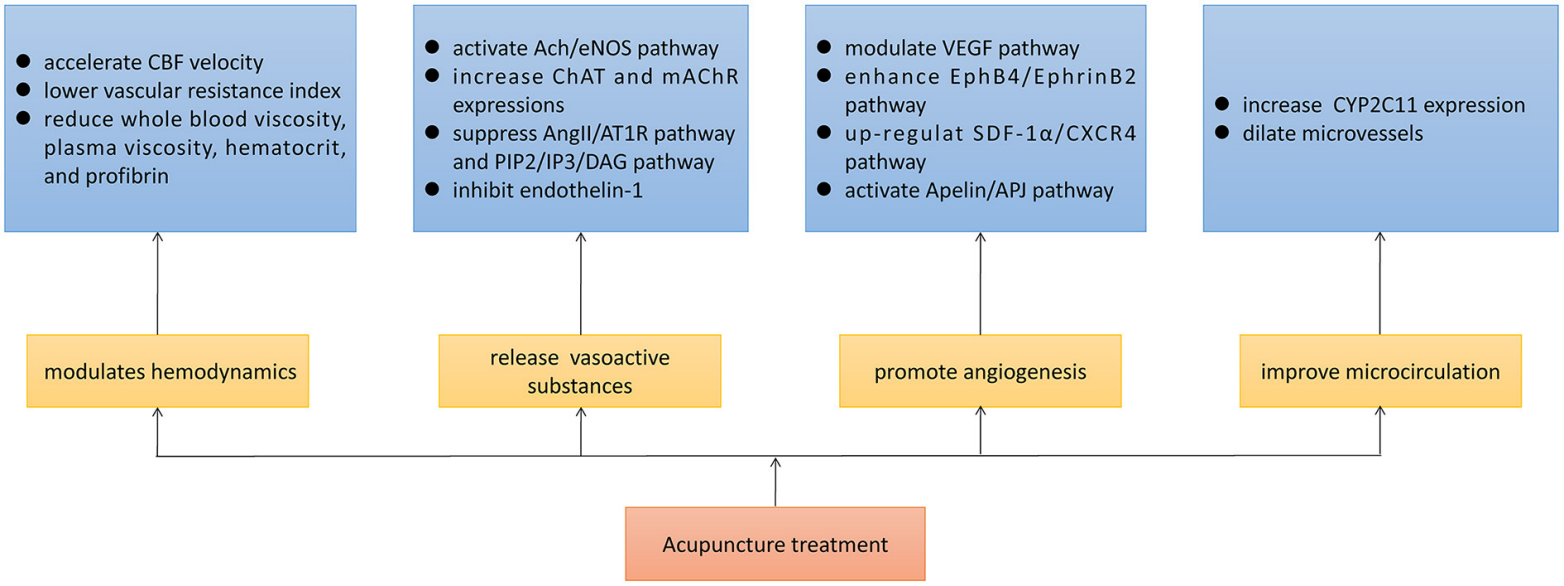
Potential mechanisms of acupuncture in enhancing cerebral perfusion of ischemic stroke.

Optimizing cerebral perfusion is the key to rescuing salvageable ischemic brain tissue. According to all evidence from the studies we have reviewed, acupuncture showed a beneficial effect on cerebral perfusion in ischemic stroke. During the initial stage of ischemic stroke, acupuncture facilitated the recovery of the CBF through modulating hemodynamic disorders and the release of vasoactive substances. During chronic ischemia, acupuncture promoted the formation of new blood vessels *via* modulating the VEGF, EphB4/EphrinB2, SDF-1α/CXCR4, and Apelin/APJ pathways. In the meantime, acupuncture improved microcirculation, enhancing energy metabolism of brain tissues and ameliorating neurological function prognosis (Figure 6).

There is substantial evidence that CBF is regulated directly by neurovascular nerves. Recently, nerve stimulation therapies, such as facial nerve stimulation (64), trigeminal nerve stimulation (65), and sphenoid palatal ganglion stimulation (66), have been reported to have beneficial effects on decreasing cerebrovascular resistance and upregulating cerebral perfusion in ischemic brain tissue. Acupuncture stimulation is a procedure involving the insertion of a fine needle into the skin or deeper tissues at specific acupoints of the body. Abundant neuroreceptors in the nerve terminals of acupoints are considered the basis of the needling sensation of patients, such as free nerve terminal muscle spindle, annular corpuscles, Kirschner's terminal ball, among others (67). After activating receptors located on the neural terminals, acupuncture signals are partly transmitted to the central nervous system, leading to modulation of brain functions (67). The most frequently used Shuigou (GV26) and Baihui (GV20) are distributed in the face and head, respectively located in the afferent sensory nerve fibers innervation range of maxillary branch and ophthalmic branch of the trigeminal nerve. Neiguan (PC6) is situated on the volar side of the wrist and belongs to the range of afferent sensory nerve fibers innervated by the median nerve. Previous studies pointed out that trigeminal nerve stimulation could cause cerebrovasodilation and enhance cerebral perfusion through the trigemino-cerebrovascular system and trigemino-parasympathetic reflex (65). Median nerve stimulation increased the regional CBF of the contralateral motor and somatosensory cortex (68). Notably, one study reported that EA to the ophthalmic branch of the trigeminal nerve enhanced CBF in the prefrontal cortex of healthy subjects (69). Further animal experiment observed that EA stimulation of acupoints on the head and face augmented the CBF in MCAO rats, while this effect disappeared when parasympathetic nerve function was blocked by unilateral vagotomy and atropine (33), suggesting that acupuncture is probably to be a potential nerve stimulation therapy to regulate CBF. However, the specific mechanism regarding how the stimulation signal of acupuncture is delivered from peripheral acupoints to the central nervous system to enhance cerebral perfusion is still unclear. Besides, whether neurovascular coupling is implicated in the regulation of CBF by acupuncture needs to be further explored.

Acupuncture is currently mainly applied clinically during the stroke rehabilitation period, adopted by the National Institutes of Health. Notably, the numerous studies that we have reviewed in this study clarified the beneficial effects of acupuncture therapy on CBF in the acute stage of ischemic stroke. As we all know, the ischemic lesion will evolve during the transport of patients to the endovascular center, and many acute stroke patients do not receive revascularization therapy like thrombolysis or thrombectomy due to the limited time window (2). Acupuncture therapy, with the advantages of non-invasive and easy operation, is available to be delivered at the prehospital stage, in the ambulance, or mobile stroke units, may have the potential to freeze the penumbra and prevent infarct growth, which will probably make the number of patients who can be treated successfully with endovascular therapy or intravenous alteplase substantially increase because the treatment time window may be prolonged. Additionally, not all patients have an opportunity to achieve early initiation of revascularization therapy on account of rigorous eligibility criteria and numerous contraindications (70), even though successful reperfusion after getting these therapeutic modalities is very likely to suffer from the no-reflow phenomenon ascribed to microcirculatory clogging (71). More importantly, the restoration of blood flow in patients received revascularization therapy may result in secondary reperfusion injury (72), a process that involves the production of reactive oxygen species, inflammation, cell apoptosis, and autophagy. Increasing evidence suggests that acupuncture can prevent the generation of excessive reactive oxygen species (73), alleviate the inflammatory response (74), and inhibit apoptosis and autophagy (75) after cerebral ischemia/reperfusion and promote repair of the injured nervous system. Therefore, acupuncture may be a promising auxiliary strategy for revascularization therapy, thereby producing cumulative effects in a synergistic form of treatment in the early phase of acute ischemic stroke.

Even though many advances have been made in further studies, there are still several questions that remain to be elucidated. Firstly, the therapeutic effects of acupuncture are influenced by plenty of variables, such as acupoint selection, stimulation frequency and intensity, and retaining needle time. Although acupuncture therapy emphasizes individuality, lacking standardized treatment regimens will hinder its promotion in clinical practice. Furthermore, there is an obvious disconnect between basic research and clinical research in the selection of acupoints and stimulation parameters, which makes the current research results unable to effectively guide clinical practice. Secondly, most studies reviewed were conducted using animal models, especially MCAO model. However, transient mechanical vascular occlusion is not a model of naturally occurring stroke and its clinical relevance in particular with respect to translational aspects is poor (76). Choosing the most appropriate stroke model might increase the extrapolation of animal data to humans. Thirdly, numerous studies apply laser Doppler flowmetry or transcranial Doppler ultrasound to monitor CBF. While these approaches are economical and convenient, they have low resolution and are prone to false positives. More advanced techniques should be applied, such as single-photon emission computed tomography, arterial spin labeling, perfusion-weighted imaging, digital subtraction angiography, CT angiography, which can more intuitively reflect the influence of acupuncture on cerebral perfusion and cerebral vascular state. Fourthly, to date, most clinical studies focus on the ameliorating effect of acupuncture on neurological dysfunction in ischemic stroke, and only a few trials pay attention to the regulating effect of acupuncture on cerebral blood flow. Besides, convincing evidence from theses clinical trials in support of acupuncture enhancing cerebral perfusion in ischemic stroke were not regarded as robust due to the methodological weaknesses, such as the use of outcome measures that were not internationally recognized, unclear methods of randomization and allocation concealment, the lack of long-term follow-up, and publication bias. Researchers need to consider carrying out more high-quality clinical trials to determine the efficacy of acupuncture used as an adjunct to standard care in ischemic stroke. What's more, fundamental issues concerning the therapeutic window, duration and mechanisms of action, and the risk of adverse effects also remain to be answered.

In summary, the above-reviewed evidence suggests that acupuncture has a positive impact on cerebral perfusion after ischemic stroke. Renewed efforts are needed to improve our understanding of acupuncture in regulating CBF and to translate these experimental findings to clinical practice.

## Author contributions

LW wrote the paper and made the pictures. LW, X-TS, YC, and N-NY searched the literature. LW, X-WH, and YC sorted out the table. J-WY, H-PL, and Q-YW revised the paper. All authors contributed to the article and approved the submitted version.

## Funding

This study was funded by the National Natural Science Foundation of China (Grant Numbers 82004479 and 81674055).

## Conflict of interest

The authors declare that the research was conducted in the absence of any commercial or financial relationships that could be construed as a potential conflict of interest.

## Publisher's note

All claims expressed in this article are solely those of the authors and do not necessarily represent those of their affiliated organizations, or those of the publisher, the editors and the reviewers. Any product that may be evaluated in this article, or claim that may be made by its manufacturer, is not guaranteed or endorsed by the publisher.
